# Perceptions, attitudes and practices regarding canine zoonotic helminthiases among dog owners in Nyagatare district, Rwanda

**DOI:** 10.1002/vms3.787

**Published:** 2022-03-22

**Authors:** Pie Ntampaka, François Niragire, Vincent Nkurunziza, Gisele Uwizeyimana, Anselme Shyaka

**Affiliations:** ^1^ Department of Veterinary Medicine University of Rwanda Nyagatare Rwanda; ^2^ Department of Applied Statistics University of Rwanda Kigali Rwanda; ^3^ Centre for One Health University of Global Health Equity Kigali Rwanda

**Keywords:** awareness, dog owners, Nyagatare, Rwanda, zoonotic helminths

## Abstract

**Background:**

Despite their importance to society, dogs are susceptible to various helminths. This study aimed to understand perceptions, attitudes and practices (PAP) regarding canine zoonotic helminthiases (CZH) among dog owners in Rwanda.

**Methods:**

A cross‐sectional study was carried out in Nyagatare district of Rwanda, where 203 dog owners were randomly selected and interviewed. To analyse this study's data, we used frequency distributions, chi‐square test of association and binary logistic regression model.

**Results:**

Overall, 75.9% and 30% of the respondents were aware of canine and human helminthiases, respectively. Around 74.4% knew that dogs source helminthiases from eating raw infected meat. Also, 74.4% knew vomiting, diarrhoea and swelling of the belly as clinical signs of the helminthiases. Around 58.6% washed hands with soap and water after handling a dog. Only 17.2% and 15.5% dewormed the dogs and treated them against ectoparasites using conventional anthelmintics and acaricides manufactured for dogs, respectively. Of all respondents, 33% held genuine perceptions, while 78.3% and 25.1% adopted positive attitudes and appropriate practices about CZH, respectively. The respondents’ educational level and length of dog ownership correlated with their perceptions and practices about CZH, respectively, while sources of information on CZH influenced their PAP of such infections. The adjusted odds ratio (OR) of having genuine perceptions and positive attitudes were more than 82% lower among those who sourced the information from neighbours & colleagues compared to those who gathered it through reading. Again, the length of dog ownership (OR = 0.37, 95% CI: 0.15–0.90) correlated with the respondent's practices about CZH.

**Conclusions:**

Only 33% and 25.1% of the respondents held genuine perceptions and adopted positive attitudes regarding CZH, respectively. The findings indicate increased zoonotic helminths in dogs, possible spillover in humans and anthelmintic resistance. Therefore, awareness campaigns are needed to upgrade dog owners’ knowledge of the zoonotic helminthiases in dogs in Nyagatare district.

## Graphical abstract

The study investigated perceptions, attitudes and practices regarding canine zoonotic helminthiases among dog owners in Rwanda.

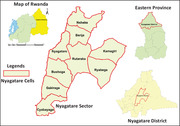

## 1 INTRODUCTION

Dogs are valued animals in human society and the role they play has gradually changed to include hunting and guarding, offering services and companionship (Beck et al., 2012; Wells, 2007). Despite their roles, they also harbour a wide range of helminths including nematodes, trematodes and cestodes (El‐Gayar, 2007; Sharma et al., 2017) and some are zoonotic such as *Ancylostoma* spp, *Toxocara canis*, *Echinococcus* spp and *Dipylidium caninum* (Jiang et al., 2017; Moskvina & Ermolenko, 2016). Some helminths are also responsible for neglected tropical infections, for example, *Ancylostoma* spp and *Echinococcus* spp  (Engels & Zhou, 2020).

Dogs get ancylostomiasis via oral ingestion or skin penetration of infective larvae. Oral transmission involves ingesting infected milk, paratenic hosts or suckling infected dams (Ballweber, 2001). In dogs, toxocariasis is transmitted via ingesting faeces or soil loaded with embryonated eggs or suckling infected dams. Pregnant bitches can also transmit the infection to foetuses (Despommier, 2003). Although *Felidae *and *Hyaenidae *can be definitive hosts for *Echinococcus* spp, domestic dogs and other *Canidae* are the main definitive hosts (Romig et al., 2017). Both herbivores and omnivores are intermediate hosts and dogs get infected when they eat meat from infected intermediate hosts (Romig et al., 2017; Vass & Nappi, 2002). Dogs can also act as aberrant intermediate hosts for  *E. multilocularis* and develop alveolar echinococcosis (Frey et al., 2017; Haller et al., 1998). Dogs develop the latter infection when they consume food, water or faeces contaminated with *E. multilocularis* eggs or autoinfect themselves when adult worms of *E. multilocularis* present in their intestines lay eggs (Frey et al., 2017; Scharf et al., 2004). Of ten species of *Echinococcus* currently known, seven are zoonotic (Thompson, 2020). Dogs develop dipylidiasis after ingesting an intermediate host (fleas) containing an egg capsule of *D. caninum*  (Despommier et al., 2019).

Humans can directly contract zoonotic helminths from animals or their products (e.g. *Trichinella spiralis*) or can indirectly get infected through invertebrate or vertebrate intermediate hosts and contaminated items such as soil and water (Youn, 2009). Humans infected with some zoonotic helminths for example *Strongyloides stercoralis* can also autoinfect themselves (Stepek et al., 2006). Humans develop *Toxocara canis* infection when they ingest items loaded with embryonated eggs while they contract hookworm (*Ancylostomatidae*) infections through oral or percutaneous routes (Despommier, 2003; Sharma et al., 2017).When they touch an infected dog, humans can accidentally ingest *Echinococcus* spp eggs and develop the infection. (Roberts et al., 2009). Humans can also develop dipylidiasis once they accidentally ingest infected dog and cat fleas (Despommier et al., 2019). If exposed cysticercoids are present on a dog tongue after grooming, the dog may also transmit dipylidiasis to people through licking them (Sapp & Bradbury, 2020).

In humans,* Ancylostoma braziliense* and *A. caninum *can cause cutaneous larva migrans while* Toxocara canis *causes visceral larva migrans and ocular larval migrans (Ballweber, 2001).

In a study conducted in dogs in Rwanda, the prevalence of *Ancylostoma* spp was 32.3% while that of *Toxocara canis* and *Ancylostoma* spp coinfection represented 1.1% (Ntampaka et al., 2021). Another study conducted in school children in Rwanda reported the prevalence of hookworms of 1.9% (Kabatende et al., 2020). Dogs suffering from toxocariasis manifest different signs including poor hair coat, general unthriftiness, slow weight increase, vomiting and diarrhoea (Ballweber, 2001; Sharma et al., 2017). The clinical picture of canine ancylostomiasis includes bloody diarrhoea and chronic anaemia in puppies and adult dogs, respectively (Sharma et al., 2017).

Although dogs suffering from dipylidiasis do not exhibit any clinical disease, some dogs can have diarrhoea and anal pruritis (Ballweber, 2001; Saini et al., 2016). Dogs having intestinal echinococcosis caused by adult worms do not show clinical signs (Haller et al., 1998). The control of canine dipylidiasis involves deworming dogs and combatting fleas, especially in households (Jiang et al., 2017). Canine ancylostomiasis, toxocariasis and echinococcosis can be controlled via prophylactic deworming (Ballweber, 2001). In addition, the prevention of echinococcosis requires preventing dogs from scavenging and predating wherever possible (Romig et al., 2017).

In Rwanda, dog owners purchase anthelmintics formulated for dogs from veterinary pharmacies and ivermectin, Ascaten‐P (contains mebendazole, piperazine citrate and praziquantel) and Univerm total (includes praziquantel, pyrantel embonate and fenbendazole) are examples of the drugs that were available at the time of this study. To the best of our knowledge, no published study has investigated the level of awareness, perceptions and practices about canine zoonotic helminthiases among dog owners in Rwanda and thus, this information is lacking. It is unknown whether dog owners are knowledgeable about the potential health risks associated with dogs, especially canine zoonotic helminthiases. A study conducted in Ghana found that 60% of dog owners were aware of canine helminthiases (Amissah‐Reynolds et al., 2016). The same study also reported that 24% knew that dogs could spread zoonotic parasites to humans and that 13% adopted appropriate practices of dog feeding (feeding them in a bowl) while 46% never dewormed their dogs. Previous studies reported a level of awareness of transmission of zoonotic parasites from dogs to humans that varied between 4.6%−10% (Kiflu et al., 2016; Panigrahi et al., 2014). Therefore, this study aimed to understand the perceptions, attitudes and practices regarding CZH among dog owners in Nyagatare district, Rwanda.

## 2 MATERIALS AND METHODS

### 2.1 Study area

This study was conducted in Nyagatare district between May and July 2019. Nyagatare is one of 30 administrative districts of Rwanda.

The district is in Eastern province of Rwanda and it borders Uganda in the North, Tanzania in the East, Gatsibo district in the South and Gicumbi district in the West. Nyagatare district is also divided into 14 administrative sectors and each one is subdivided into administrative cells and as a whole, the district covers 106 cells. First, Nyagatare district was purposively selected because it is a district dedicated to farming and has the highest cattle population in Rwanda. Given that many dairy cattle farmers also keep guard dogs, we wanted to assess whether the farmers are aware of the potential health risks associated with dogs, especially canine zoonotic helminthiases. Second, Nyagatare sector was selected as it was home to the largest dog population in the district. Given Nyagatare city is also among the six secondary cities in Rwanda; it is rapidly growing and dog ownership has been on the rise (World Bank Group, 2017).

**FIGURE 1:**
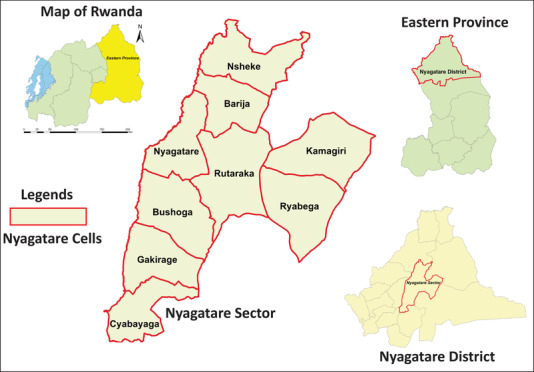
The map of study area. Fig. 1 shows Nyagatare administrative sector (red boundaries) and its nine administrative cells, namely, Cyabayaga, Gakirage, Bushoga, Nyagatare, Rutaraka, Ryabega, Kamagiri, Barija and Nsheke

The current study covered six of the nine administrative cells of Nyagatare sector including Nyagatare, Barija, Nsheke, Ryabega, Rutaraka and Bushoga. The remaining three cells were home to a very small dog population and were not included in this study. The map of Nyagatare sector and the study area is shown (Figure 1). Figure 1 shows Nyagatare administrative sector (red boundaries) and its nine administrative cells. The map was generated using ArcGis10.2 software based on shapefiles available at DIVA‐GIS (Hijmans, 2012).

### 2.2 Study design and sample size

We conducted a cross‐sectional study involving structured questionnaire‐based interviews with dog owners living in Nyagatare sector. In 2019, Nyagatare sector‐registers indicated a population of 800 dogs. Although the dog population would be slightly more than the households owning dogs, we assumed that every household owned one dog. Thus, this study considered 800 households to represent a reasonable population size of dog owners for calculating the sample size of participants. By the time of this study, there was no published work on perceptions, attitudes or practices of canine zoonotic helminthiases in Rwanda, thus a theoretical proportion of 50% was used to maximise the sample size (Bartlett et al., 2001). Allowing a maximum estimation error of 5% and a confidence intervals of 95%, the sample size was determined using Cochran formula for sample size for categorical data (Bartlett et al., 2001). Therefore, a representative sample of 203 households was required for this study. Using the listing of dogs, the first dog was randomly chosen and the other 202 dogs were picked out by systematically selecting every third dog on the list.

Considering the population of 800 and sample size of 203, *i* = 800/203 = 3.9. In a circular sampling, we typically round down that is to 3. With the help of local leaders, the owners of the selected dogs were subsequently visited at their households for interviews.

### 2.3 Data collection

A questionnaire consisting of both closed and open questions was specifically designed for this study and it was pretested before interviewing the respondents. During the pretest, the questionnaire was administered to seven dog owners who were randomly contacted and interviewed for judging it: the raised weaknesses were worked on. The questions focused on PAP regarding CZH among dog owners in Nyagatare administrative sector. Each interview lasted for about 15 minutes and the respondents provided answers in Kinyarwanda and these were translated back in English before cleaning and analysing the data. During data collection and analysis, each respondent's data records were identified with a unique code.

### 2.4 Data analysis

All the participants’ answers to survey questions were classified as either correct (scored with 1) or wrong (scored with 0) (Alrubaiee et al., 2020; Della Polla et al., 2020; ul Haq et al., 2012).The participants’ perceptions were measured using six questions: (i) *how dogs can develop helminthiases;* (ii) *how dogs can contract helminthiases*; (iii) *can people contract helminthiases from dogs*; (iv) *how people can contract helminthiases via dogs*; (v) *are you aware of clinical signs of canine helminthiases* and *(vi) can ectoparasites transmit helminthiases to dogs*. Respondents’ perceptions were classified as either *genuine*, if they could provide correct answers to more than 50% of the questions, that is at least 3 (out of 6) correct answers or *misguided* perceptions otherwise (Alrubaiee et al., 2020; Della Polla et al., 2020; ul Haq et al., 2012).

Respondents’ attitudes were evaluated using three questions that asked: (i) what they would do to observe hygiene after handling a dog; (ii) *what they would do if their dogs are suffering from helminthiasis* and (iii) *how they would handle a dog after he/she becomes a source of a human helminthiasis*. The respondents’ attitudes were considered *positive*, if they could provide correct answers to at least two items and negative attitudes otherwise.

The practices about dog‐spread zoonotic helminthiases were assessed with six items that asked the participants: (i) *how they fed their dogs*; (ii) *whether they had dewormed their dogs*; (iii) *whether they diagnosed helminthiases before deworming their dogs;* (iv) *the kind of anthelminthics their dogs received;* (v) *whether they treated their dog(s) against ectoparasites* and (vi) *the timing of their dogs’ treatment against ectoparasites*. The practice was considered as *appropriate*, if the respondent gave correct answers to at least 3 of 6 questions and as *inappropriate* practice otherwise.

It followed that all the PAP variables were dichotomous. Collected data were organised, summarised and analysed using IBM Statistical Package for Social Sciences (IBM SPSS) Statistics version 23. A univariate analysis summarised data with frequency distributions and bivariate analysis conducted a series of chi‐square test of association between the PAP variables and the potential explanatory variables. Finally, a multivariable binary logistic regression analysis was performed to identify the key factors affecting the PAP regarding canine zoonotic helminthiases.

### 2.5 Results

#### 2.5.1 Sociodemographic characteristics of respondents

Overall, 203 participants were recruited into this study and they were of different background in terms of age, sex, education, occupation and length of dog ownership. Of all respondents, 100% were dog owners and 93.1% kept dogs for security reason. Nearly 62.1% did farming and 61.6% sourced information about canine helminths from colleagues and neighbours (Table 1).

**TABLE 1:** Sociodemographic characteristics of the respondents

Respondent's characteristics	Frequency	Percentage
Age group		
18–35	54	26.6
36–45	100	49.3
>45	49	24.1
Total	203	100.0
Sex		
Male	150	73.9
Female	53	26.1
Total	203	100.0
Educational level		
Non‐formal education	21	10.3
Primary School	88	43.4
Secondary education	65	32.0
Tertiary education	29	14.3
Total	203	100.0
Occupation		
Farming	126	62.1
Other	77	37.9
Total	203	100.0
Number of dogs kept		
One	112	55.2
More than one	91	44.8
Total	203	100.0
Length of dog ownership		
<1 year	41	20.2
1–3 years	109	53.7
>3 years	53	26.1
Total	203	100.0
Reason for keeping dogs		
Security	189	93.1
Companion	14	6.9
Total	203	100.0
Source of information about dog helminths		
Reading	49	24.1
Professionals	29	14.3
Neighbours & colleagues	125	61.6
Total	203	100.0

### 2.6 Respondents’ perceptions about canine zoonotic helminthiases

Approximately 75.9% of the respondents perceived that dogs could develop helminthiases and 74.4% were aware that dogs can get infected through eating raw infected meat. Only 8.9% recognised that dogs can contract the infections through ingesting ectoparasites (fleas). The percentage of respondents who perceived that dogs could transmit helminthiases to people and the correct routes through which humans can contract such infections represented 30% each. Vomiting, diarrhoea and swelling of belly were the known clinical signs accounting for 74.4%. The proportion of the respondents who held genuine perceptions on CZH represented 33% (Table 2). The results in Table 3 indicate that the proportion of dog owners who had genuine perceptions was higher in those who completed secondary school (34.3%) compared to other educational levels.

**TABLE 2:** Perceptions on dog‐spread zoonotic helminthiases among respondents

	Frequency	Percentage
Know dogs can develop helminthiases		
No	49	24.1
Yes	154	75.9
Total	203	100.0
Know how dogs can contract helminthiases		
No	49	24.1
Yes	154	75.9
Total	203	100.0
Know people can contract helminthiases from dogs		
No	142	70.0
Yes	61	30.0
Total	203	100.0
Know how people can contract helminthiases from dogs		
No	142	70.0
Yes	61	30.0
Total	203	100.0
Know clinical signs of canine helminthiases		
No	52	25.6
Yes	151	74.4
Total	203	100.0
Know accidental ingestion of fleas can be a source of canine helminthiases		
No	185	91.1
Yes	18	8.9
Total	203	100.0
Proportion of the respondents with genuine or misguided perceptions		
Genuine	67	33.0
Misguided	136	67.0
Total	203	100

**TABLE 3:** Associations of the respondents’ perceptions, attitudes and practices with sociodemographic characteristics

	Perceptions		Attitudes		Practices	
Factors	Genuine^†^	*p* Value	Positive^‡^	*p* Value	Appropriate^§^	*p* Value
Age group						
18–35	31.3	0.442	25.2	0.675	29.4	0.663
36–45	43.3		50.3		51.0	
> 45	25.4		24.5		19.6	
Sex						
Male	74.6	0.867	73.6	0.850	82.4	0.112
Female	25.4		26.4		17.6	
Education						
Non‐formal	6.0	0.001	8.2	0.045	9.8	<0.001
Primary	32.8		41.5		27.5	
Secondary	34.3		33.3		29.4	
Tertiary	26.9		17.0		33.3	
Occupation						
Farming	53.7	0.086	57.2	0.007	47.1	<0.001
Other	46.3		42.8		52.9	
Length of dog keeping						
< 1 year	25.4	0.188	22.6	0.255	31.4	0.028
1–3 years	44.8		52.2		39.2	
> 3 years	29.9		25.2		29.4	
Sources of information						
Reading	42.6	<0.001	28.9	<0.001	37.3	<0.001
Professionals	23.5		17.6		31.4	
Neighbours & colleagues	33.8		53.5		31.4	

^†^ Percentage of respondents with genuine perceptions regarding CZH.

^‡^ Percentage of respondents who adopted positive attitudes toward CZH.

^§^ Percentage of respondents who adopted appropriate practices of CZH.

Further, the proportion of dog owners who held genuine perceptions was higher in respondents who sourced information on CZH through personal reading (42.6%) compared to other sources of information.

#### 2.6.1 Attitudes of the respondents towards controlling and preventing helminthiases in humans and dogs

Around 58.6% of the respondents had correct attitudes to dog handling, while 90.1% would take correct attitudes to a dog after he/she transmits helminthiasis to a person. Only 21.2% would have correct attitudes towards a dog suffering from helminthiasis and the proportion of the respondents who took positive attitudes towards CZH was 78.3% (Table 4). Further, the proportion of dog owners who took positive attitudes towards CZH was higher in those who finished primary school (41.5%) compared to other educational categories. It was also higher in those who did farming (57.2%) as well as those who received information on CZH from neighbours and colleagues (53.5%) compared to those who did other business and received information from other sources, respectively (Table 3).

**TABLE 4:** Attitudes of respondents towards controlling and preventing helminthiases in humans and dogs

	Frequency	Percentage
What would you do to observe hygiene after handling a dog?		
Nothing (wrong)	78	38.4
Wash hands with water (wrong)	6	3.0
Wash hands with water and soap (correct)	119	58.6
Total	203	100.0
What would you do to your dog if it becomes a source of human helminthiasis?		
Treat it (correct)	183	90.2
Kill it (wrong)	6	3.0
Chase it from home (wrong)	7	3.4
Do nothing (wrong)	7	3.4
Total	203	100.0
What would you do when a dog suffers from helminthiasis?		
Do nothing (wrong)	98	48.3
Treat it with herbal medicine (wrong)	62	30.5
Treat it with conventional anthelminthics (correct)	43	21.2
Total	203	100.0
Proportion of the respondents with positive or negative attitudes		
Positive	159	78.3
Negative	44	21.7
Total	203	100

#### 2.6.2 Practices relating to the control of canine zoonotic helminthiases

Of all respondents, 33.5% and 29.6% adopted appropriate practices of feeding and of dog deworming, respectively. Only 5.4% of those who dewormed their dogs (*n* = 60) adopted appropriate practices about the diagnosis of helminthiasis before deworming. Around 17.2% and 15.8% adopted appropriate practices regarding the treatment against helminths and ectoparasites, respectively. Ascaten‐P^®^ was the most used combination (12.3%), followed by Ivermectin (3.4%) and Univerm total^®^ (1.5%) was the least used combination (Table 5). The proportion of the respondents who adopted appropriate practices regarding CZH accounted for 25.1% (Table 6). The results in Table 5 show that the proportion of dog owners who adopted appropriate practices about CZH was higher in those who received tertiary education (33.3%) compared to those who completed lower educational levels. Again, it was higher in those who were involved in activities other than farming (52.9%). It was also higher in dog owners who acquired information on CZH through person reading (37.3%) compared to those who got information from other sources.

**TABLE 5:** Practices of the control of canine zoonotic helminthiases among the respondents

	Frequency	Percentage
How do you feed your dog(s)?		
Scavenging dog (inappropriate)	94	46.3
Feed it in utensils (appropriate)	68	33.5
Feed it on bare ground (inappropriate)	41	20.2
Total	203	100.0
Did you deworm your dog(s)?		
No (inappropriate)	143	70.4
Yes (appropriate)	60	29.6
Total	203	100.0
Did you diagnose canine helminthiasis before applying anthelminthics?		
No (inappropriate)	49	24.1
Yes (appropriate)	11	5.4
Do not deworm (inappropriate)	143	70.5
Total	203	100.0
What kind of anthelminthics did your dog receive?		
Conventional drugs_Ascaten‐P,^†^ Ivermectin, Univerm total^‡^ (appropriate)	35	17.2
Herbal medicine (inappropriate)	25	12.3
Did not deworm (inappropriate)	143	70.5
Total	203	100.0
Do you treat your dog(s) against ectoparasites?		
No (inappropriate)	171	84.2
Yes (appropriate)	32	15.8
Total	203	100.0
When do you treat your dog(s) against ectoparasites?		
Do it when I spray cattle or other livestock (inappropriate)	94	46.3
Do it only for dogs (appropriate)	32	15.8
Do not treat the dogs against ectoparasites (inappropriate)	77	37.9
Total	203	100.0
Proportion of the respondents with appropriate or inappropriate practices		
Appropriate	51	25.1
Inappropriate	152	74.9
Total	203	100

^†^ A combination of mebendazole, piperazine citrate, praziquantel.

^‡^ A combination of praziquantel, pyrantel embonate, fenbendazole.

**TABLE 6:** Logistic regression analyses of the factors associated with PAP among dog owners in Nyagatare, Rwanda (*n* = 203)

		Perceptions	Attitudes	Practices
Variable	Category	OR (95% CI)	OR (95% CI)	OR (95% CI)
Educational background				
	Non‐formal education	*1.00 (reference)*	*1.00 (reference)*	*1.00 (reference)*
	Primary school	2.11(0.58–7.68)	2.52(0.85–7.48)	0.67(0.19–2.37)
	Secondary education	2.12(0.56–8.04)	1.94(0.59–6.33)	0.67(0.18–2.51)
	Tertiary education	5.38(1.17–24.34)	3.00(0.47–19.18)	2.40(0.55–10.41)
Respondent’ occupation				
	Farming	*1.00 (reference)*	*1.00 (reference)*	*1.00 (reference)*
	Other	0.78(0.35–1.69)	1.92(0.78–4.72)	1.30(0.56–3.04)
Respondent’ source of information				
	Reading	*1.00 (reference)*	*1.00 (reference)*	*1.00 (reference)*
	Professionals	0.78(0.29–2.08)	1.58(0.15–16.54)	1.83(0.67–5.04)
	Neighbours & colleagues	0.18(0.08–0.40)	0.15(0.04–0.53)	0.34(0.14–0.81)
Length of dog keeping				
	<1 year	*1.00 (reference)*	*1.00 (reference)*	*1.00 (reference)*
	1–3 years			0.37(0.15–0.90)
	>3 years			0.69(0.26–1.82)
Constant		0.64	5.84	0.96
		*	**	***

* Hosmer‐Lemeshow goodness of fit test statistic: 2.47, *p *= 0.872.

** Hosmer‐Lemeshow goodness of fit test statistic: 3.65, *p *= 0.819.

*** Hosmer‐Lemeshow goodness of fit test statistic 6.27, *p *= 0.509.

#### 2.6.3 Chi‐square test of associations of the respondents’ perceptions, attitudes and practices about canine zoonotic helminthiases

The respondents’ educational level and sources of information on canine zoonotic helminthiases correlated with their perceptions, attitudes and practices of such infections in Nyagatare.

Besides, their occupation correlated with attitudes and practices and the length of dog ownership also positively influenced the respondents’ practices of canine zoonotic helminthiases in Nyagatare (Table 3).

#### 2.6.4 Logistic regression analyses

We modelled the probability of responding correctly to at least 50% of the questions using a multivariable binary logistic model. The results in Table 6 show that the OR of having genuine perceptions about canine zoonotic helminthiases for those who completed tertiary education were more than five times higher than the odds for those who had no formal education. Also, the respondents’ sources of information on canine zoonotic helminths correlated with their perceptions, attitudes and practices regarding the infection. Specifically, the adjusted OR of having genuine perceptions and positive attitudes were more than 82% lower among those who sourced the information from neighbours and colleagues compared to those who gathered it through reading. Again, the length of dog ownership (OR = 0.37, 95% CI: 0.15–0.90) correlated with the respondent's practices regarding the zoonotic helminthiases in dogs. Specifically, the odds of adopting appropriate practices of canine zoonotic helminthiases were 63% lower among those who kept dogs for 1–3 years than the odds for those who owned dogs for less than a year.

## 3 DISCUSSION

This study aimed to understand the perceptions, attitudes and practices regarding canine zoonotic helminthiases among dog owners in Nyagatare district, Rwanda. To our knowledge, this study is the first to assess the PAP of dog owners about CZH in Rwanda.  Overall, < 35% of the respondents held genuine perceptions, while 78.3% and 25.1% adopted positive attitudes and appropriate practices of CZH, respectively. The methods of sourcing information on CZH among the respondents correlated with their PAP regarding such infections. Further, their educational level and length of dog ownership influenced their perceptions and practices of CZH, respectively. These findings can help veterinary and medical leaders prioritise interventions aimed at upgrading the knowledge of CZH among the dog owners and the residents of Nyagatare district at large.

We found that 75.9% of the respondents perceived that dogs can contract helminthiases and the percentage was higher than 60% and 46.7% reported in Ghana and Brazil, respectively (Amissah‐Reynolds et al., 2016; Katagiri & Oliveira‐Sequeira, 2008). Thirty percent (30%) identified that dogs spread helminthiases to humans and the proportion was higher than 10% and 7.4% reported in India and Nigeria, respectively (Panigrahi et al., 2014; Ugbomoiko et al., 2008). However, it was lower than 55% revealed in Canada (Stull et al., 2012).

The methods of getting information on CZH among the respondents might have negatively impacted the proportion of those who held genuine perceptions. Our respondents mainly sourced the information from friends and colleagues, while the respondents interviewed by Stull et al. in Canada mainly received information from veterinarians. Maybe, when dog owners sought information from veterinarians, they received accurate information on CZH while they got little information when they received it from colleagues and friends. In Rwanda, veterinary training started a few years ago; the number of veterinarians is still insufficient and the penetration of veterinary services is not commendable (Brown et al., 2020; World Organisation for Animal Health, 2019). Such circumstances would predispose dog owners and farmers towards sourcing animal health information from their peers rather than veterinarians. The 61.6% of our respondents who acquired information on canine zoonotic helminths from colleagues and friends was higher than 48% found in Ethiopia (Kiflu et al., 2016). Again, 14.3% of those who sourced it from professionals, including veterinarians, was lower than 18.6% (Kiflu et al., 2016). Although veterinary education in Ethiopia started many years ago compared to that of Rwanda (Brown et al., 2020), this indicates that sources of animal health information vary between locations. Sourcing information regarding CZH from colleagues and friends can result in sharing inaccurate information and predisposing dog owners to adopting inappropriate practices against such infections.

We found that 58.6% of the respondents would wash hands with water and soap after handling a dog and this was lower than 78.8% who washed their hands after touching a dog in Ethiopia (Kiflu et al., 2016). Hand washing is a good practice of mitigating zoonotic infections (Stull et al., 2012; Wong & Lee, 2019) and adopting it (e.g. using water and soap) would remove dirt, including dog‐spread helminth eggs from human hands. Our findings showed that 33.5% of the respondents fed their dogs in utensils and the figure was higher than 13% reported in Ghana (Amissah‐Reynolds et al., 2016). Feeding dogs on the bare ground can predispose them to helminthiases. For instance, canine toxocariasis can be transmitted through ingesting soil loaded with embryonated eggs (Despommier, 2003).

We found that 17.2% of the respondents dewormed their dogs using conventional anthelmintics. This percentage was higher than that reported in Ethiopia, where not a single respondent applied conventional anthelmintics (Kebede, 2019). It was however lower than 50.9% reported in Ghana (Johnson et al., 2015). Around 12.3% of our respondents dewormed their dogs with herbal medicine and the percentage was lower than 39% in a study conducted in Ethiopia (Kebede, 2019). Medicinal plants can treat human and animal (e.g. dogs) helminthiases (Mali & Mehta, 2008).

Rwandan traditional healers use *Phytolacca dodecandra* vernacularly known as Umuhoko as a taenifuge and *Clutia abyssinica* vernacularly known as Umutarishonga as ascarifuge (Ramathal & Ngassapa, 2001). In this study, only 5.4% dewormed their dogs after the diagnosis. The application of anthelmintics without performing the diagnosis could progressively lead to anthelminthic resistance (Pullola et al., 2006). Approximately 15.8% treated their dogs against ectoparasites using acaricide formulations manufactured for dogs. The rest did (or not) spray theirs after treating cattle or small ruminants (sheep/goats) against ectoparasites. The application of acaricide manufactured for cattle in dogs could fail the product (Coles & Dryden, 2014). Our findings are of public health importance and adopting one health approach would help control human helminths. This study has some limitations: its design did not allow to determine the level of knowledge of the dog owners about zoonotic helminths in dogs rather their perceptions. Future studies should ascertain the dog owners’ level of knowledge and the prevalence of zoonotic helminths in the dog population.

## 4 CONCLUSION

Our findings show that 33% and 25.1% of the respondents held genuine perceptions and adopted positive attitudes regarding CZH, respectively. The findings indicate increased zoonotic helminths in dogs, possible spillover in humans and anthelmintic resistance. Therefore, awareness campaigns are needed for upgrading dog owners’ knowledge about zoonotic helminths in dogs in Nyagatare district.

## AUTHOR CONTRIBUTIONS

Pie Ntampaka: Conceptualization, Methodology, Validation, Visualization, Writing original draft. François Niragire: Data curation, Formal analysis, Methodology, Writing review & editing. Vincent Nkurunziza: Conceptualization, Data curation, Investigation, Writing review & editing. Gisele Uwizeyimana: Conceptualization, Investigation, Writing review & editing. Anselme Shyaka: Methodology, Validation, Visualization, Writing review & editing.

## CONSENT STATEMENT

The owner of this case has consented to the disclosure of this case's information.

### PEER REVIEW

The peer review history for this article is available at https://publons.com/publon/10.1002/vms3.787

## Data Availability

The data that support the findings of this study are available from the corresponding author upon reasonable request.
